# A Dynamical Role for Acetylcholine in Synaptic Renormalization

**DOI:** 10.1371/journal.pcbi.1002939

**Published:** 2013-03-14

**Authors:** Christian G. Fink, Geoffrey G. Murphy, Michal Zochowski, Victoria Booth

**Affiliations:** 1 Department of Physics, University of Michigan, Ann Arbor, Michigan, United States of America; 2 Department of Physics & Astronomy and Neuroscience Program, Ohio Wesleyan University, Delaware, Ohio, United States of America; 3 Molecular & Behavioral Neuroscience Institute, University of Michigan, Ann Arbor, Michigan, United States of America; 4 Department of Molecular & Integrative Physiology, University of Michigan, Ann Arbor, Michigan, United States of America; 5 Biophysics Program, University of Michigan, Ann Arbor, Michigan, United States of America; 6 Departments of Mathematics and Anesthesiology, University of Michigan, Ann Arbor, Michigan, United States of America; Université Paris Descartes, Centre National de la Recherche Scientifique, France

## Abstract

Although sleep is a fundamental behavior observed in virtually all animal species, its functions remain unclear. One leading proposal, known as the synaptic renormalization hypothesis, suggests that sleep is necessary to counteract a global strengthening of synapses that occurs during wakefulness. Evidence for sleep-dependent synaptic downscaling (or synaptic renormalization) has been observed experimentally, but the physiological mechanisms which generate this phenomenon are unknown. In this study, we propose that changes in neuronal membrane excitability induced by acetylcholine may provide a dynamical mechanism for both wake-dependent synaptic upscaling and sleep-dependent downscaling. We show *in silico* that cholinergically-induced changes in network firing patterns alter overall network synaptic potentiation when synaptic strengths evolve through spike-timing dependent plasticity mechanisms. Specifically, network synaptic potentiation increases dramatically with high cholinergic concentration and decreases dramatically with low levels of acetylcholine. We demonstrate that this phenomenon is robust across variation of many different network parameters.

## Author Summary

The function of sleep is one of the greatest mysteries in contemporary neuroscience. Nearly every species of animal requires it, yet we do not know why. One idea, known as the *synaptic renormalization hypothesis*, suggests that waking results in a global increase in the strengths of connections in the brain, a phenomenon which is unsustainable because stronger connections consume more energy and take up more space. The function of sleep, according to this hypothesis, is to downscale or “renormalize” connection strengths. While mounting experimental evidence confirms that sleep-dependent synaptic downscaling does occur, we still do not know what biophysical mechanism *causes* it. In this paper, we show computational results which indicate that the neuromodulator acetylcholine may have a key role to play in sleep-dependent synaptic downscaling. If confirmed experimentally, these findings will help to unravel the mystery of sleep.

## Introduction

Sleep is crucial for normal cognitive function as evidenced by the many cognitive impairments associated with chronic sleep loss [1], [2]. A leading proposal for the function of sleep, called the synaptic renormalization hypothesis, posits that sleep is required to maintain synaptic balance in the brain [3], [4]. According to this hypothesis, waking experiences result in the net potentiation of many brain circuits, leading to both increased energy consumption and heightened demand for space by the potentiated synapses. In order to conserve energy and space, sleep induces a period of large-scale synaptic downscaling. Sleep is therefore “the price we pay for plasticity” [5].

Multiple lines of empirical evidence supporting the synaptic renormalization hypothesis have recently emerged [6]–[10], including *in vivo* studies finding increased slope of evoked LFP/EEG responses after wakefulness and decreased slope following sleep in rats [11] and humans [12]. Furthermore, increasing evidence supports a link between synaptic depotentiation during sleep and slow wave activity (SWA) [13], which is the pattern of electroencephalograph (EEG) activity observed during non-rapid eye movement (NREM) sleep in mammals and birds which features increased power in the delta band (0.5 to 4 Hz). Various studies have shown that SWA in NREM sleep locally increases in brain areas that exhibit potentiation during prior wakefulness [14]–[16], suggesting that SWA may function to maintain synaptic homeostasis.

Exactly how synaptic downscaling is induced during sleep is an open question. One suggestion is that the repeated alternation of depolarized “up” states, reflecting the simultaneous activity of many neurons, and hyperpolarized “down” states, reflecting fewer active neurons, observed to occur at approximately 1 Hz during SWA may induce long-term depression (LTD) of synapses [17], [18]. Another possibility is that the reduction of brain-derived neurotrophic factor (BDNF) during sleep [5], [6] might enable synaptic depression. Similarly, it is not clear exactly why synapses might exhibit net potentiation during wakefulness, though it has been suggested that the processing of sensory signals or the formation of new memories may inevitably lead to synaptic upscaling [4].

A further hypothesis is that differences in the neuromodulators available during waking and NREM sleep states may contribute to the opposing effects of wakefulness and NREM sleep on neuronal potentiation levels [5]. Waking is characterized by high levels of noradrenaline, serotonin, histamine and acetylcholine in cortex, while all these neurotransmitters are at low levels during NREM sleep [19], [20]. The low levels of these neuromodulators during sleep has led to the idea that this alters molecular mechanisms underlying spike-timing dependent plasticity (STDP) so that sleep favors synaptic depotentiation [21]. Although some investigation has been done into the effects of various neuromodulators on STDP [22], these mechanisms remain poorly understood. The effects of neuromodulators upon other forms of plasticity may also contribute to synaptic renormalization [23], [24].

In the present study, we build upon previous work to develop a new theory for synaptic downregulation during NREM sleep that highlights a role for differing cortical network dynamics during wake and NREM sleep states. This theory relies upon previous findings showing that acetylcholine (ACh) modulates the phase-dependence of neural responses in cortex [25], [26]. When ACh is more available, as in the awake state, most cortical neurons display phase-independent firing in response to synaptic input: they fire soon after receiving excitatory input regardless of their activity when the input arrives (Type I). In contrast, when ACh is less available, as during NREM sleep, cortical neurons display phase-dependent firing in response to synaptic input: whether they fire sooner or later after receiving an excitatory input depends on how long it has been since they last fired (Type II). As we and others have shown previously, the increased flexibility of exact firing times in response to input that occurs with low ACh concentration better enables pre- and post-synaptic cells to synchronize their activity, thereby increasing synchronized activity in cortical networks [27]–[29]. While ACh has many diverse effects in the brain [30], [31], here we focus on these dynamical effects of cholinergic modulation.

Our new theory concerns the effect of increased synchronized network activity during NREM sleep on the strength of synaptic connections. In particular, we posit that although this increase in synchronized network activity strengthens some individual synaptic connections, it weakens others. Further, and critically, this weakening is more pronounced when an animal is experiencing NREM sleep (more synchronized activity) than when an animal is awake (less synchronized activity). Supporting this novel hypothesis, we show that a computational model employing these dynamic, physiologically-plausible mechanisms is fully able to account for synaptic renormalization during NREM sleep.

## Results

**Figure 1:**
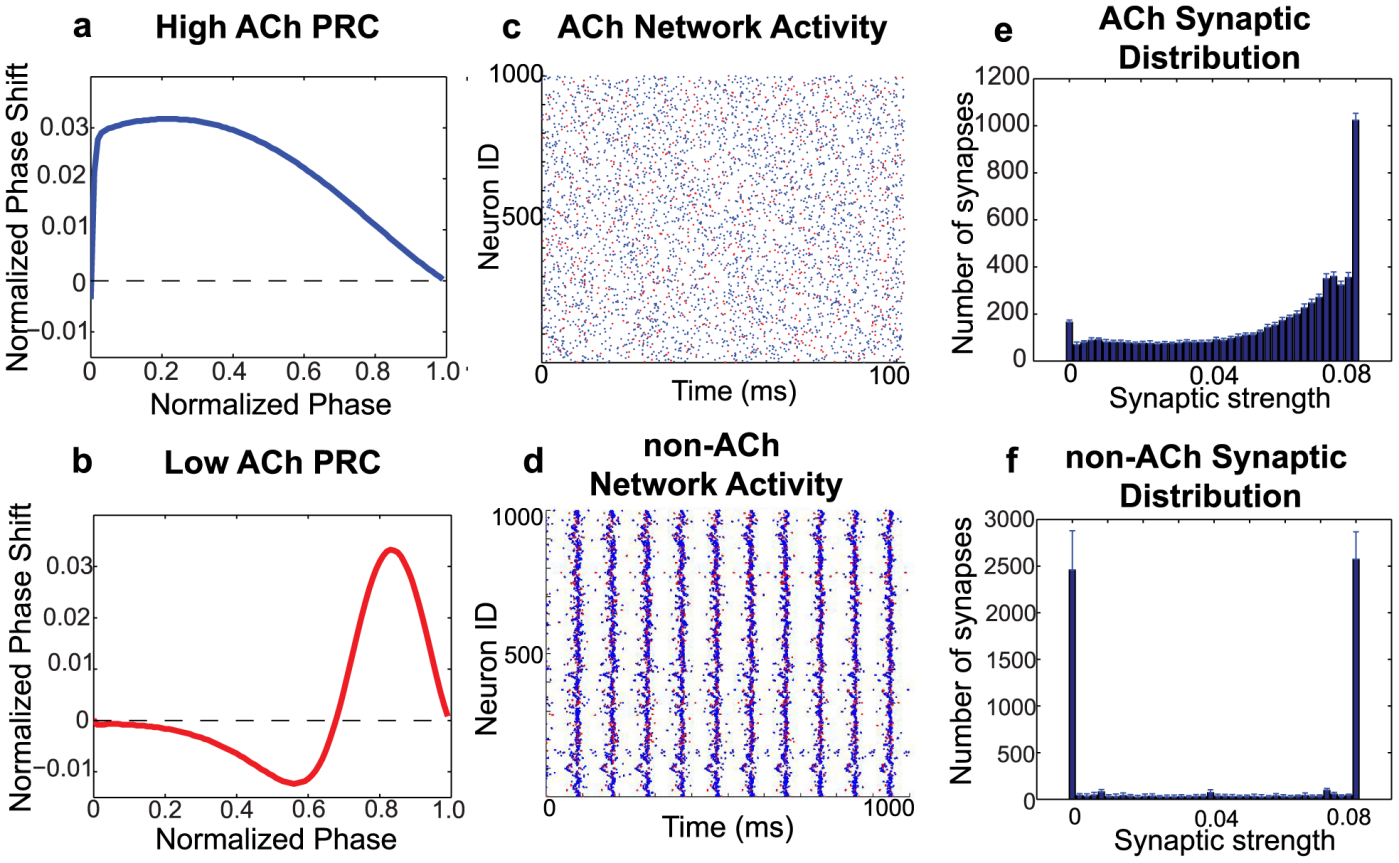
Effects of acetylcholine on phase response curves, network synchrony, and overall network synaptic potentiation in 1000-cell cortical neuronal network models. (a,b) Phase response curves of individual neurons with (a) high simulated ACh concentration and (b) low simulated ACh concentration. (c, d) Raster plots of the activity of a model cortical network with (c) high and (d) low ACh concentration. Blue (Red) dots represent spikes of excitatory (inhibitory) neurons. Note the higher synchronization in the network with low cholinergic modulation compared to the network with high cholinergic modulation. (e) Average final distributions of synaptic strengths for a typical high-ACh network, with a network potentiation value of 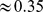. (f) Average final distribution of synaptic strengths for a typical low-ACh network. This distribution constitutes a much lower network potentiation value (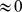) due to a greater proportion of synapses with zero synaptic strength values. In panels (c)–(f), the re-wiring probability was 0.60 and 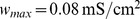. Panels (e) and (f) represent histograms averaged over ten different network initializations.

We simulated the effects of ACh on synaptic potentiation in cortical networks consisting of 1000 neurons (20% of which were inhibitory). Each neuron was described by a recently-developed cortical pyramidal cell model [26] that was motivated by experimentally measured effects of ACh [25]. In this model, simulated cholinergic modulation blocks a slow, low-threshold M-type potassium current that induces spike frequency adaptation. Blockade of this current modulates the response properties of modeled neurons as measured by the phase response curve (PRC). With low ACh levels, the neuronal PRC displays phase regions where spike timing is delayed and where it is advanced, categorized as Type II PRC [28], [29]. High ACh levels produce only advances in spike timing regardless of the phase of perturbation, resulting in Type I PRC (see Fig. 1).

Switching PRCs of synaptically coupled neurons from Type II to Type I has been shown to dramatically affect the synchronization of neuronal networks. Specifically, simulated large-scale neuronal networks whose cells have Type II PRCs have been shown to synchronize much better than neuronal networks composed of cells with Type I PRCs [32]. This effect can be explained heuristically by the fact that neurons with Type II PRC are in some sense “more flexible” than those with Type I PRC, since neurons with Type II PRC can advance *and* delay their spike firing in response to synaptic input [28], [29]. More rigorous mathematical analysis has shown that in the weak coupling limit, the emergence of stable synchronous dynamics depends upon a stability criterion known as the H-function, which is constructed from the odd part of the neuronal PRC [33]. Such analysis has shown that while the emergence of a phase delay region in the PRC is sufficient to promote stable synchrony, it is not necessary–a PRC which is entirely positive but skewed toward late phase can also elicit highly synchronous dynamics [34]. The designations “Type I” and “Type II” therefore constitute two poles of a spectrum of neuronal response properties. The PRC framework has been used to explain why cholinergic modulation has a dramatic effect upon the synchronization of simulated cortical networks, with low ACh concentration (which induces more Type II-like PRC) leading to much higher network synchrony than high ACh concentration (which induces more Type I-like PRC) [25]–[27].

We investigated how the differential effects of ACh on network synchrony influenced overall network synaptic potentiation when synaptic strengths evolved according to a spike-timing dependent plasticity (STDP) rule. In our network simulations, synaptic strength values were initialized to an intermediate value and then allowed to evolve, according to the STDP rule, over the interval 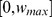 (see Materials and Methods for simulation details). We quantified the steady state distribution of synaptic strength values with a measure of “network potentiation,” calculated as a scaling of the mean equilibrium synaptic weight. The values of this network potentiation measure range from −1 for maximally weakened networks (all synaptic strength values go to 0) to +1 for maximally strengthened networks (all synaptic strength values go to 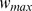). We investigated the effects of network connectivity by varying synaptic connection architecture using the Watts-Strogatz small-world paradigm [35]. With this method, each neuron was initially connected to a fixed number of its nearest neighbors, and then a certain proportion of these connections were re-wired to synapse onto randomly-selected cells in the network. The proportion of connections which were re-wired was specified by the *re-wiring probability*. Since both maximum synaptic strength and network connectivity structure are known to dramatically influence neuronal network dynamics, we explored a wide range of values for 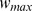 and the re-wiring probability to ensure the robustness of our results.

### Dynamical effects of acetylcholine on network synchronization and potentiation

**Figure 2:**
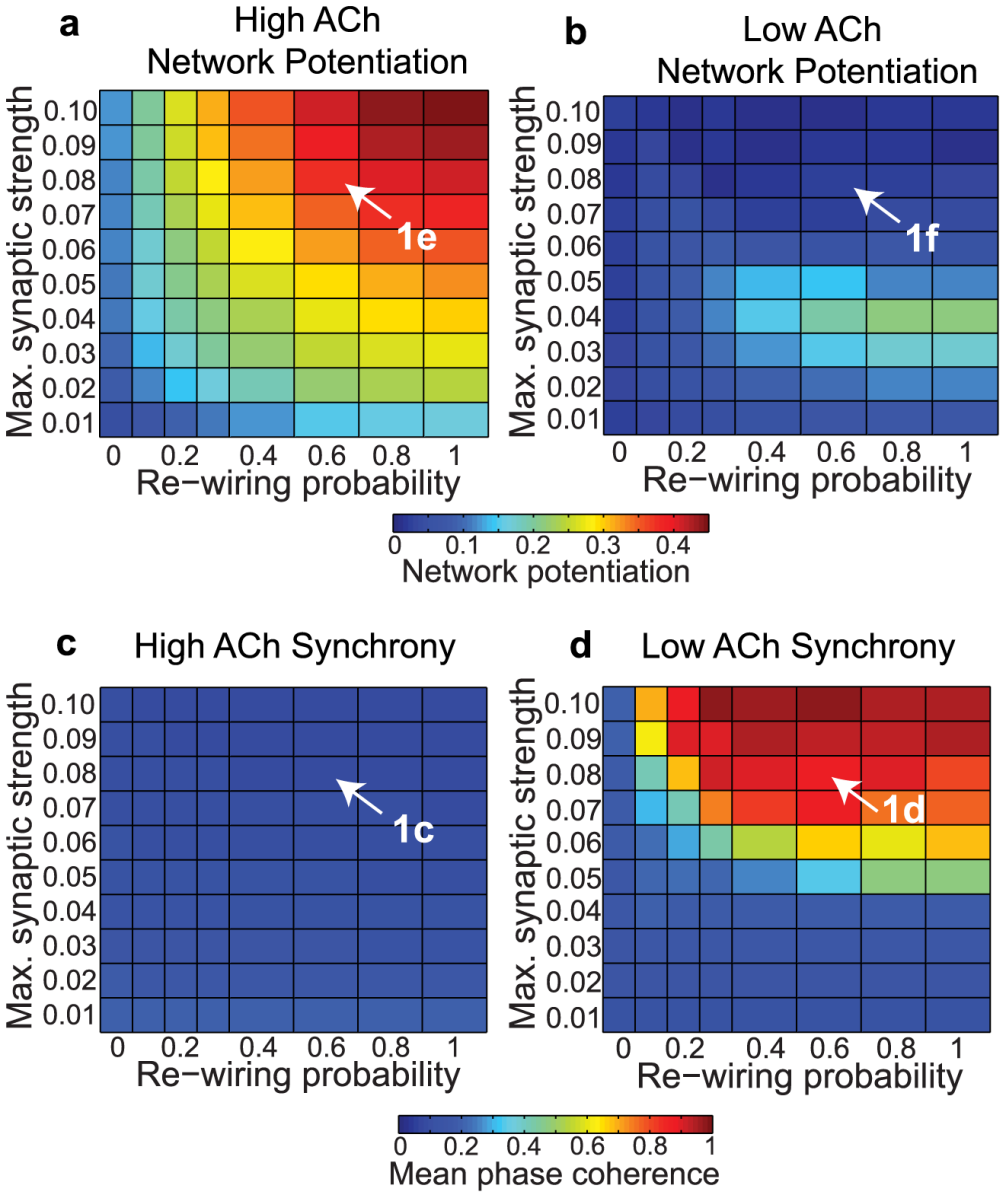
Effects of acetylcholine on network potentiation and synchronization for varied network parameters with an additive STDP rule. (a,b) Network potentiation as a function of re-wiring probability (controlling randomness of network connections, x-axis) and maximum synaptic strength (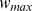, y-axis) for model cortical networks both with (a) high and (b) low simulated cholinergic modulation. Note the much greater potentiation of high-ACh networks for virtually all network parameters, and especially for 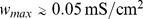. (c,d) Network synchrony, as measured by mean phase coherence, as a function of re-wiring probability and 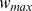 for networks with (c) high and (d) low simulated cholinergic modulation. All results represent averages over ten randomly-initialized network simulations. Arrows indicate network parameters which gave rise to panels c, d, e, and f in Fig. 1.

High simulated cholinergic modulation switched neuronal PRCs from Type II to Type I (Fig. 1 a,b), inducing a decrease in network synchronization (Fig. 1 c,d) that affected the steady state distributions of synaptic strengths (Fig. 1 e,f). The synaptic strength distribution of the high-ACh network was heavily skewed toward maximal synaptic weight, reflecting higher network potentiation. On the other hand, the distribution of the low-ACh network was more symmetric, with about half the synapses at the maximal value and the majority of remaining synapses at zero strength. These results were robust to variations in maximal synaptic strength and network connectivity architecture (Fig. 2 a,b). Network potentiation values for high-ACh networks exceeded those for low-ACh networks for almost all combinations of re-wiring probability and 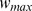.

Differences in network potentiation were especially pronounced for 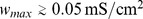, at which values the network potentiation dropped to approximately zero in low-ACh networks for all values of the re-wiring probability (Fig. 2b). Interestingly, this drop in network potentiation coincided with the transition from asynchronous to synchronous activity in low-ACh networks (Fig. 2d). On the other hand, the robustly high levels of potentiation observed in high-ACh networks (Fig. 2a) corresponded to completely asynchronous activity for all network parameters (Fig. 2c). Our simulations therefore counterintuitively showed that synchronous network dynamics led to relatively lower network potentiation than asynchronous network dynamics.

**Figure 3:**
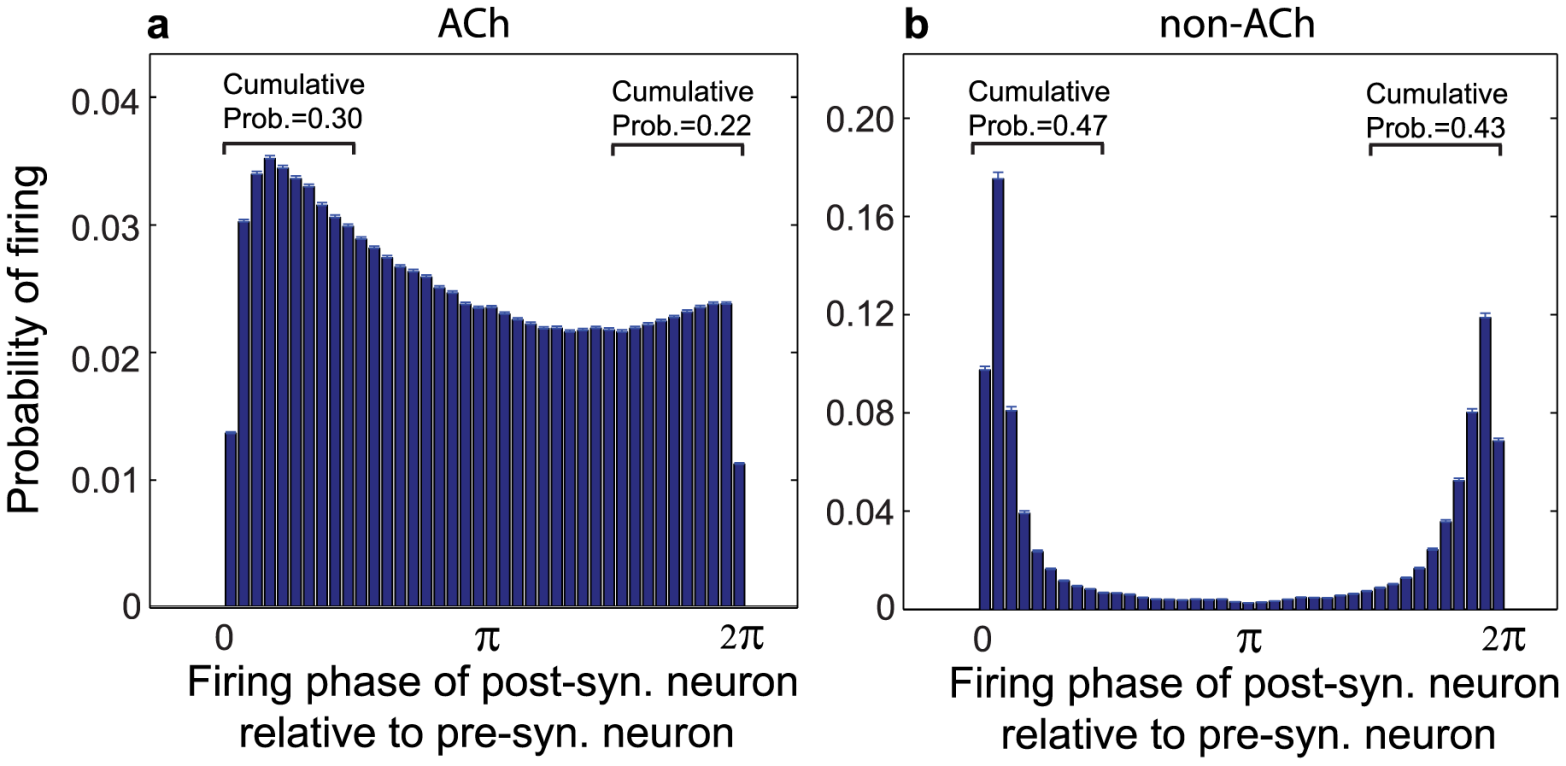
Structure of neuronal firing of pre- and post-synaptic cell pairs in high-ACh and low-ACh cortical networks. (a,b) Spike-timing histogram of phases of post-synaptic cell firing relative to pre-synaptic cell firing in the model cortical network both with (a) high and (b) low simulated cholinergic modulation. These plots were constructed by averaging the spike-timing histograms of all pre-post pairs throughout the entire network. (a) In high-ACh networks, post-synaptic cells were much more likely to fire shortly *after* (as opposed to shortly before) pre-synaptic spikes, as evidenced by the fact that the cumulative probability of firing within the interval 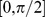 (0.30) was substantially larger than the cumulative probability of firing within the interval 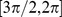 (0.22). (b) In low-ACh networks, post-synaptic spike timings were more balanced between shortly preceding and shortly succeeding pre-synaptic spikes, leading to much lower network potentiation via the STDP rule. Both histograms were computed from simulations in which the re-wiring probability was 0.60 and 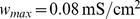. Note the different scales on the y-axes.

Since STDP requires correlated firing to potentiate the connection between two neurons, one might expect that asynchronous network activity should induce no net change in network potentiation, rather than the overall increased potentiation we observed. Further analysis of pre- and post-synaptic cell pairs uncovered an important statistical structure of the neuronal firing patterns in the cholinergically-modulated networks: post-synaptic neurons throughout the network were more likely to fire shortly after their pre-synaptic neurons rather than shortly before (Fig. 3a). Thus, pre-post spike time differences landed in the positive portion of the STDP curve more frequently than in the negative portion of the STDP curve, resulting in increased potentiation of the network as a whole.

On the other hand, the relatively lower network potentiation observed in networks with low cholinergic modulation was due to post-synaptic neurons firing right before their pre-synaptic partners much more frequently (Fig. 3b). This effect occurred because the bursts of activity in low-ACh networks constrained all neurons to fire within very short time windows, forcing pre-synaptic neurons to directly compete with one another to induce common post-synaptic partners to fire. As a result, roughly half the pre-post spike time differences fell in the positive portion of the STDP curve, and the other half fell in the negative portion, leading to nearly symmetric and highly polarized final distributions of synaptic strengths (as in Fig. 1f).

**Figure 4:**
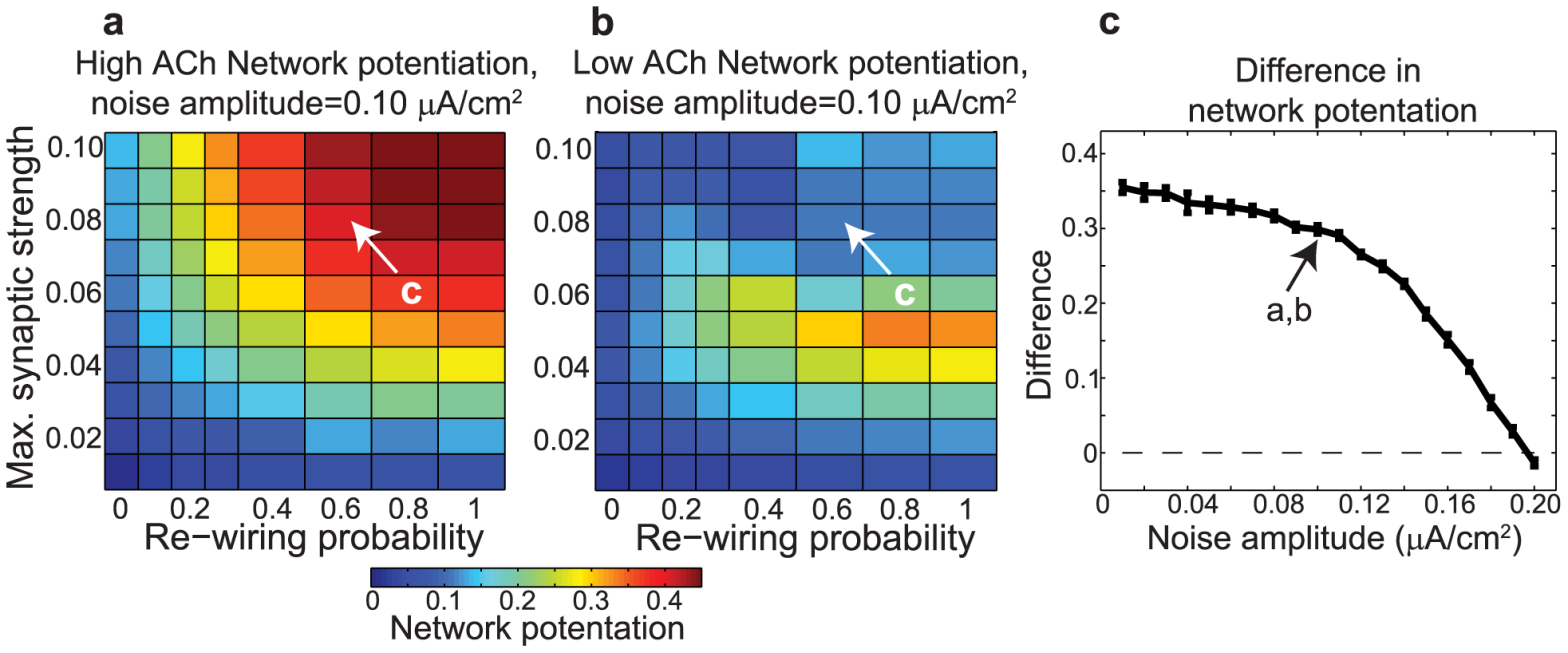
Effects of noise amplitude on the difference in network potentiation between networks with high and low cholinergic modulation. (a,b) Network potentiation as a function of re-wiring probability (x-axis) and maximum synaptic strength 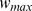 (y-axis) for networks with (a) high and (b) low cholinergic modulation, with noise amplitude fixed at 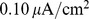. Note that high-ACh networks exhibited much greater potentiation than low-ACh networks for 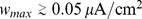. (c) Difference in network potentiation between high- and low-ACh networks as a function of noise amplitude for the network parameters indicated by arrows in panels (a) and (b).

It should be noted that we tested this result for robustness against noise by adding Gaussian-distributed noise with a temporal correlation of 100 ms (the approximate inter-spike interval of the slowest-firing neurons) to the external constant current driving individual neurons. We found that even for a noise amplitude as high as 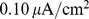, we still observed much greater potentiation in high-ACh networks than in low-ACh networks for a large range of network parameters (Fig. 4a,b). This noise amplitude was large relative to the driving currents for both high-ACh networks (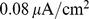) and low-ACh networks (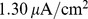). Furthermore, we found that if we chose one set of network parameters and progressively increased the noise amplitude, the difference between network potentiation in high- and low-ACh networks did not disappear until the noise amplitude reached 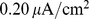 (Fig. 4c).

**Figure 5:**
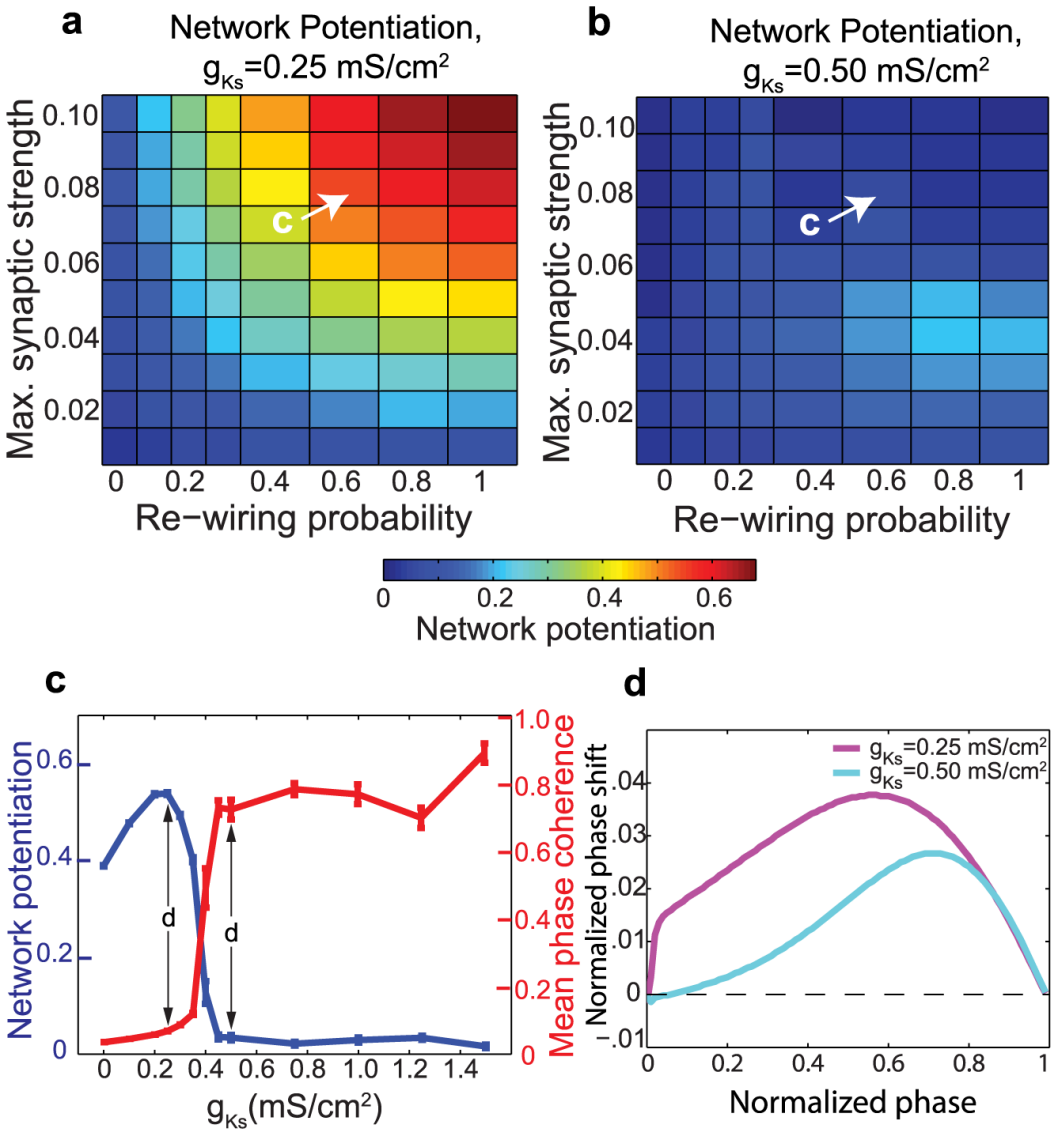
Effects of varying the slow potassium conductance, 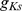
 **, upon network potentiation and PRC.** (a,b) Examples of network potentiation as a function of 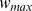 and re-wiring probability for 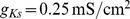 and 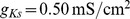. (c) Network potentiation and synchronization (as measured by mean phase coherence) as a function of 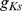 for the network parameters indicated in (a) and (b). (d) Phase response curves corresponding to 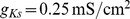 and 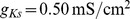.

Since acetylcholine levels vary dramatically in cortex, we investigated how sensitively our results depended upon acetylcholine levels, which dramatically influence PRC shape. Cholinergic modulation was modeled by varying the slow potassium conductance 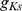 which decreases with increasing levels of acetylcholine. Fig. 5 depicts the dependence of network potentiation upon 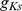 (in all other plots, 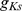 is set to 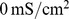 to simulate high ACh concentration and 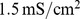 to simulate low ACh concentration). Figs. 5a,b show examples of the network potentiation plotted as a function of network parameters for two different 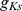 values. Note how 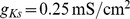 results in much greater network potentiation than 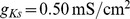 for most network parameters. Fig. 5c shows that for representative network parameters, network potentiation and network synchrony undergo sharp phase transitions as 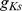 increases. The phase transition in synchrony (which induces the phase transition in network potentiation) is well explained by the transition in PRC shape depicted in Fig. 5d. As 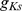 increases, the neuronal PRC is shifted to the right and, crucially, the positive slope at phase zero is attenuated while the negative slope at later phase is not. This is consistent with the idea that network synchrony stabilizes when the odd part of the PRC, known as the H-function, switches the sign of its slope at phase zero [29], [36].

**Figure 6:**
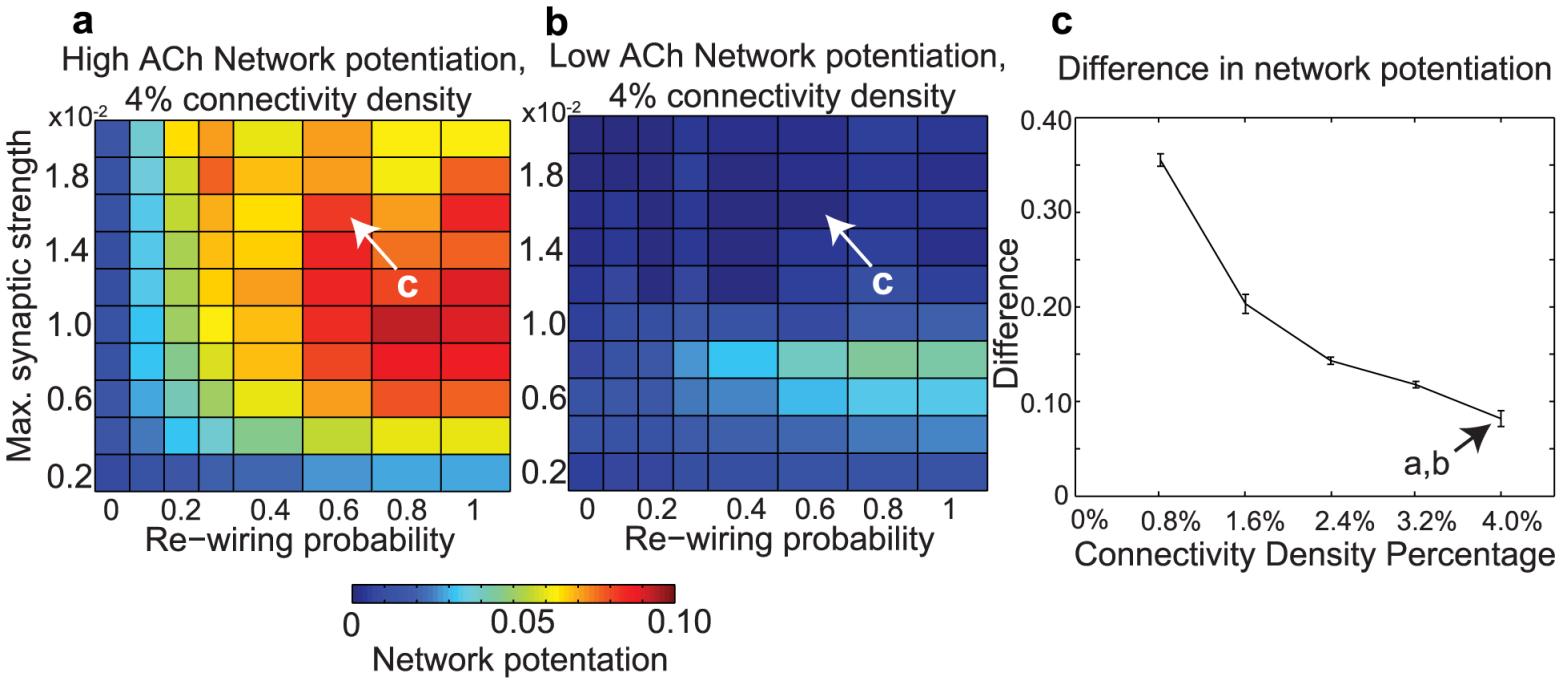
Effects of connectivity density upon network potentiation. (a,b) Network potentiation of high-ACh and low-ACh networks with 4.0% connectivity density. Network potentiation is displayed as a function of 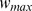 and re-wiring probability, as in Fig. 2. Note the difference in scale between these plots and Fig. 2. (c) Difference between high-ACh and low-ACh network potentiation values as a function of connectivity density for networks with parameters analogous to those indicated by arrows in panels (a) and (b). In order to investigate similar regimes of network excitability, we decreased 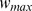 in proportion to the increase in connectivity density.

We also tested our results for robustness to connectivity density by increasing the radius of connectivity in our network simulations (see the description of the Watts-Strogatz small world network paradigm detailed in Materials and Methods). High-ACh networks showed greater overall potentiation than low-ACh networks for a wide range of connectivity densities (0.8% to 4.0% connectivity), though sparser connectivity led to greater differences in network potentiation (Fig. 6).

**Figure 7:**
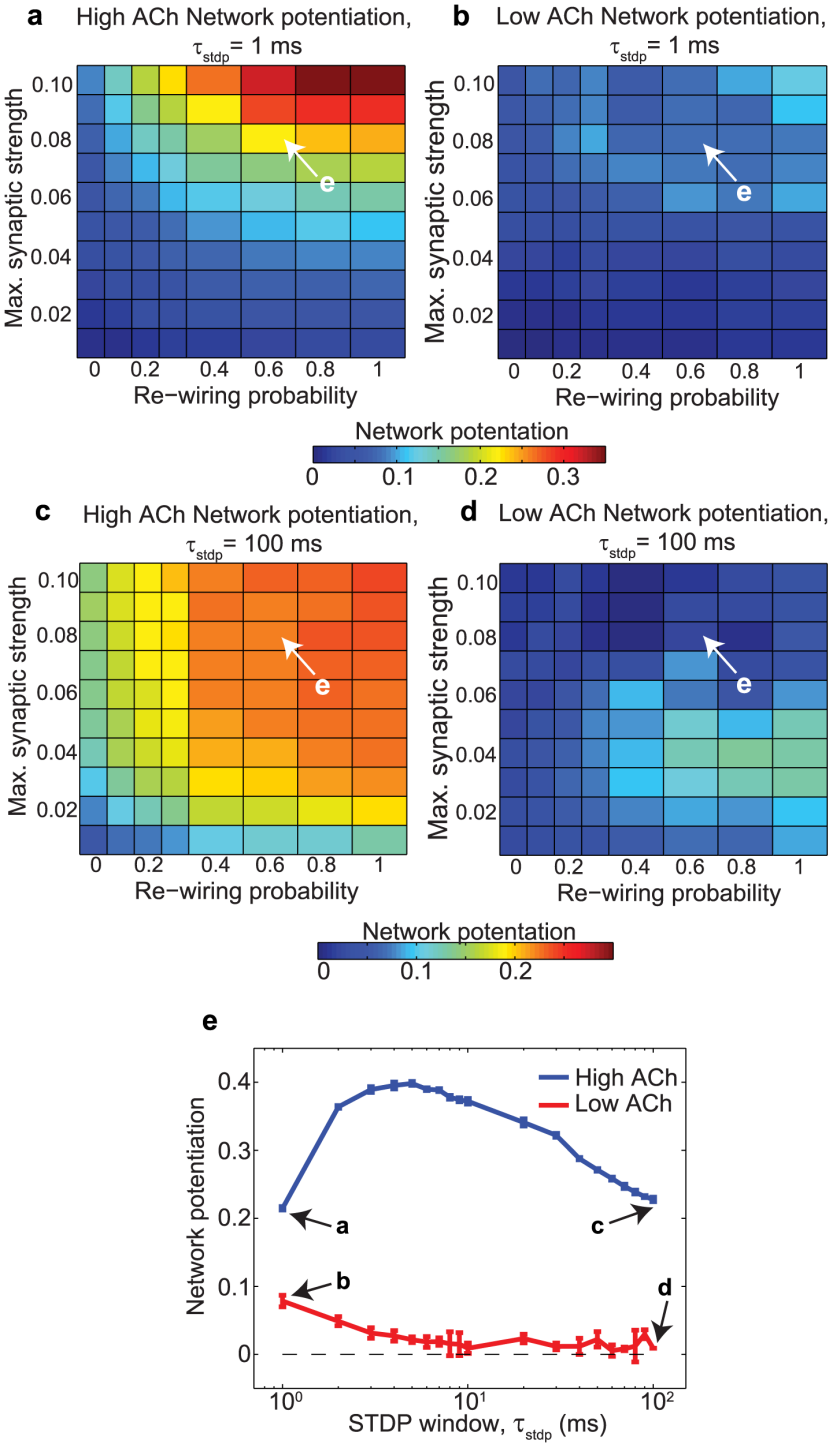
Effects of the modulation of the STDP window, 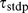
 **, upon network potentiation.** (a,b) Network potentiation of high-ACh and low-ACh networks as a function of 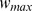 and re-wiring probability for 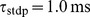. (c,d) Network potentiation of high-ACh and low-ACh networks as a function of 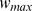 and re-wiring probability for 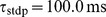. (e) Network potentiation of both high-ACh and low-ACh networks as a function of 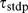, with 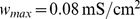 and a re-wiring probability of 0.60.

We tested the results for robustness to frequency modulation by varying the duration of the STDP window, 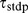. We used this approach rather than directly modulating neuronal frequency because network effects made it difficult to elicit a wide range of average firing frequencies. In Fig. 7, 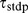 was varied from 1 ms to 100 ms (the default value throughout this study was 10 ms). High-ACh networks exhibited much higher network potentiation than low-ACh networks for all values of 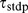.

**Figure 8:**
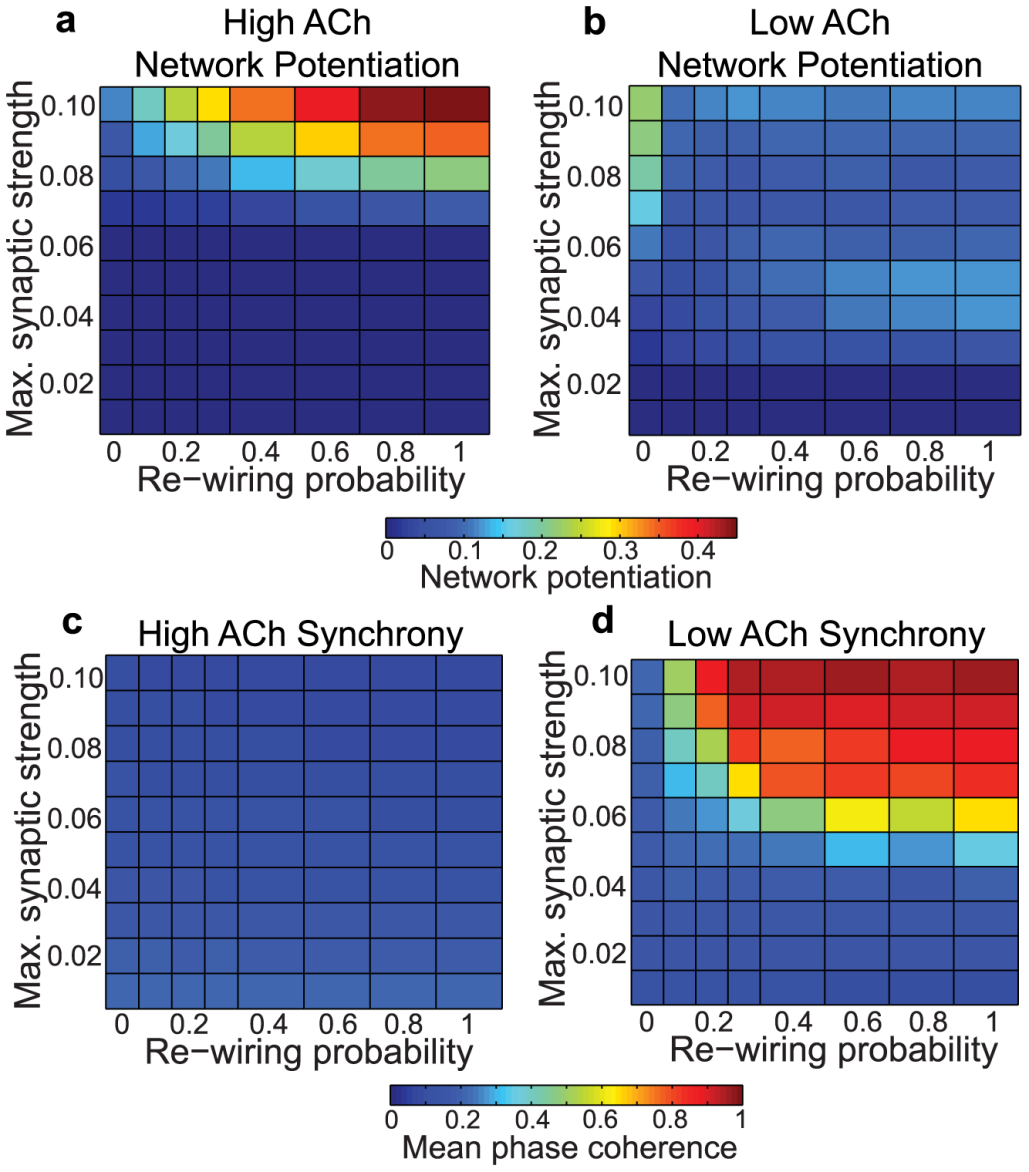
Effects of acetylcholine on network potentiation (a,b) and synchronization (c,d) for varied network parameters with an asymmetric STDP rule that favors LTD over LTP. STDP parameters were 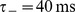, 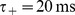, 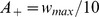, and 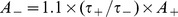.

**Figure 9:**
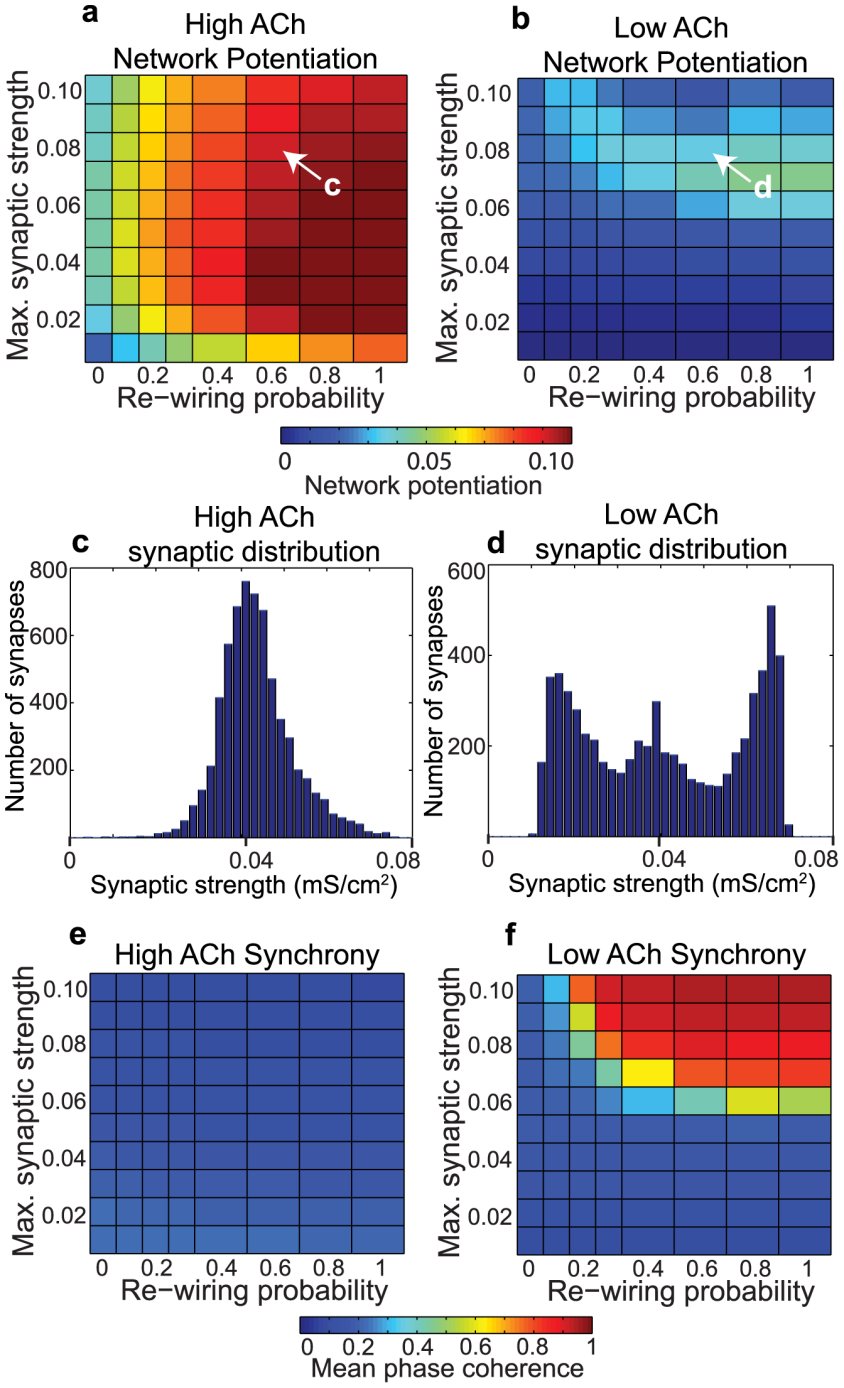
Effects of acetylcholine on network potentiation (a,b) and synchronization (e,f) for varied network parameters with a multiplicate (weight-dependent) STDP rule. As found in previous studies, the distribution of synaptic weights is not bimodal (c,d). Note the difference in scale between network potentation plots for the multiplicative STDP rule (a,b) versus the additive STDP rule (Fig. 2a,b). In both cases, high ACh concentration results in significantly greater network potentiation than low ACh concentration.

Finally, several studies have shown that the equilibrium distribution of synaptic weights in a network subject to STDP strongly depends upon the mathematical form of the STDP rule. For example, some have suggested that the integral of the LTD portion of the STDP curve should be greater than the LTP portion of the curve in order to maintain network potentiation at reasonable levels [37], [38]. We explored this STDP formulation by using an asymmetric STDP rule in which the integral of the LTD curve was ten percent greater than the integral of the LTP curve. The results of these simulations, shown in Fig. 8, are qualitatively similar to our main results in Fig. 2. Others have pointed out that “multiplicative” (weight-dependent) STDP rules tend to produce qualitatively different synaptic weight distributions than “additive” STDP rules [39]. Indeed, the polarized synaptic weight distributions shown in Fig. 1 are the typical result of an additive STDP rule [40], [41], and when we switched to a multiplicative rule we obtained more unimodal distributions (Fig. 9). For both STDP rules, we observed that high ACh led to significantly greater network potentiation than low ACh (Figs. 2 and 9), though the effect was more pronounced for the additive rule (Fig. 2a,b) than for the multiplicative rule (Fig. 9a,b).

### Switching acetylcholine levels in a heterogeneous network

The above results pertained to networks with homogeneous connectivity distributions in the sense that all synapses could achieve the same maximal strength, and long-range network connections did not preferentially target any particular neurons. Such homogeneity certainly does not exist in the brain [42], [43]. Therefore, we explored effects of cholinergic modulation on synaptic potentiation in the presence of network connectivity heterogeneities. A question of particular interest was whether ACh-induced changes in synaptic plasticity affect all connections in the network to the same extent. To address this question, we considered a network of 1000 neurons with an embedded cluster of 50 neurons. The maximal synaptic strength values (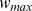) of connections originating from cells within the cluster were two times greater than for the surrounding network. Additionally, while the number of outgoing connections per neuron was the same for both the cluster and the rest of the network, a fixed fraction of out-going synaptic connections from surrounding cells preferentially targeted the cluster and vice versa. Thus, in the network, a small number of connections originated within the cluster and projected outside the cluster, while a larger number of connections originated outside the cluster and projected to the cluster (see Materials and Methods for more details).

**Figure 10:**
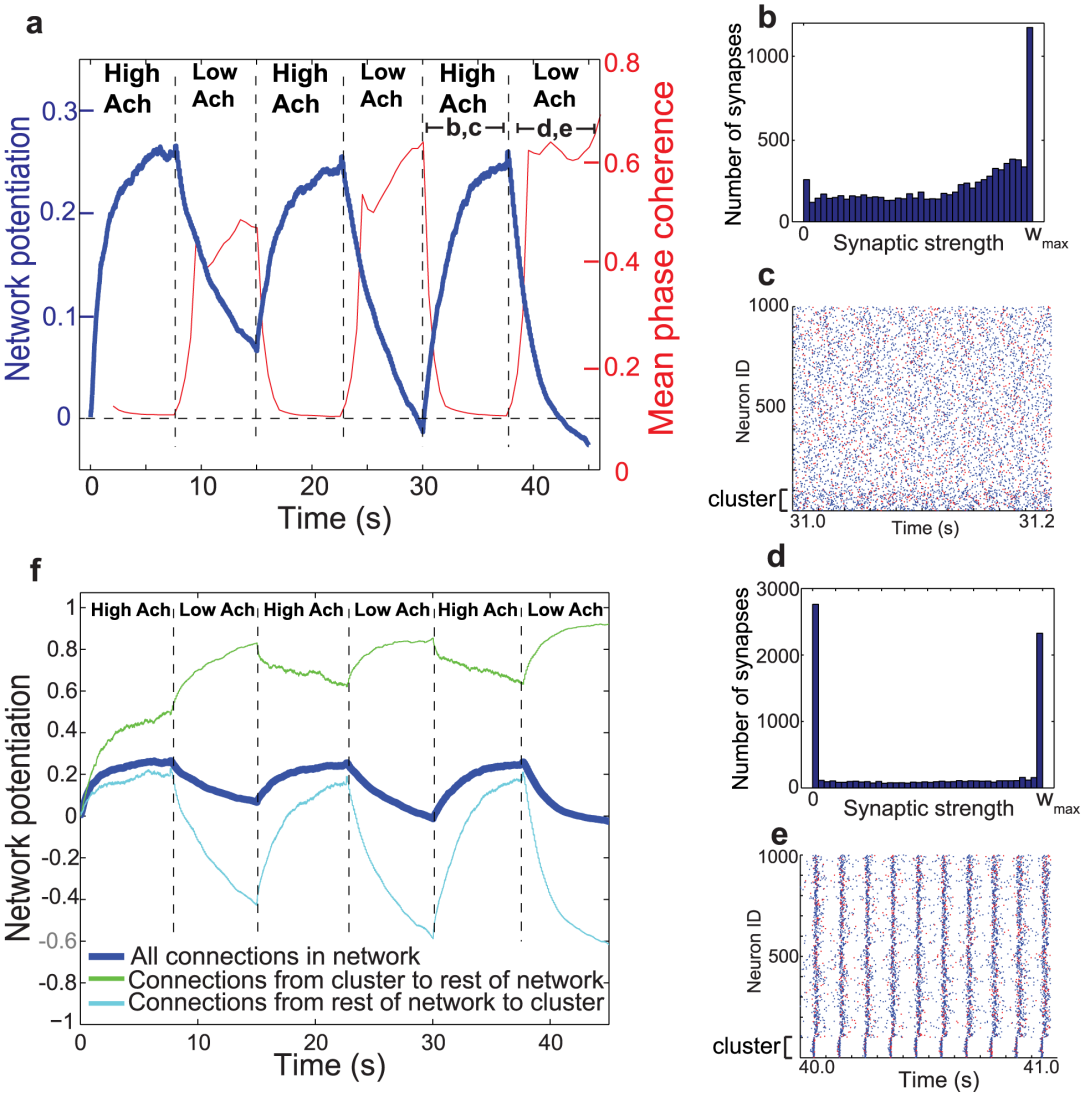
Effects of alternately switching between high and low levels of acetylcholine in a cortical network with an embedded cluster. (a) Network potentiation and synchronization (as measured by mean phase coherence) of the cortical network as a function of time as the level of ACh was alternated between high and low levels (different intervals are demarcated by dashed lines). (b) Distribution of synaptic strength values at the end of the last high-ACh interval. (c) Representative raster plot of network activity during the last high-ACh interval. The first 50 neurons comprise the cluster. (d) Distribution of synaptic strength values at the end of the last low-ACh interval. Note how the number of connections whose synaptic strength went to 0 is greater than the number that went to 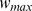. (e) Representative raster plot of network activity during the last low-ACh interval. Note how the tight bursting of the cluster drove activity in the rest of the network. (f) Network potentiation computed from distributions of synaptic weights for all synaptic connections (heavy blue curve, as shown in (a)), for synapses originating in the cluster and projecting outside the cluster (green curve), and for synaptic connections originating outside the cluster and projecting to the cluster (light blue curve). During the low-ACh intervals, the connections originating outside the cluster and projecting to the cluster showed extreme relative depotentiation due to the driving of the rest of the network by the cluster.

In this heterogeneous network, we alternately switched between the high and low acetylcholine concentration (simulating waking and NREM sleep, respectively), and found that such switching induced immediate and dramatic changes in network synchrony and potentiation (Fig. 10a). As in the homogeneous networks, we found that the asynchronous dynamics induced by high cholinergic modulation resulted in relatively high network potentiation (Fig. 10b,c), but we found that the depotentiating effects of low acetylcholine levels were even more pronounced than in homogeneous networks. Fig. 10a shows that the network potentiation measure actually dipped below zero for two low-ACh intervals, implying that the number of connections whose synaptic strength went to 0 exceeded the number that reached 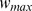 (Fig. 10d).

This enhanced depotentiating effect resulted from the dynamical interplay between the cluster and the rest of the network. As shown in Fig. 10e, under low levels of acetylcholine the cluster tended to fire in synchronized bursts, which drove the rest of the network to respond by firing noisy bursts. The relative firing times of the surrounding network relative to the cluster resulted in potentiation of connections originating in the cluster and projecting outside the cluster, and depotentiation of connections originating outside the cluster and projecting to the cluster (see the “low Ach” intervals in Fig. 10f). Since there were more connections originating outside the cluster and projecting into the cluster than vice versa, strong overall network de-potentiation occurred.

Fig. 10f demonstrates another striking feature of this network: the small subset of connections projecting from the cluster to the surrounding network remains at very high potentiation levels throughout cholinergic switching. Furthermore, this set of connections collectively *increases* in strength during epochs when ACh is low, in contrast to the collective weakening exhibited by connections in the rest of the network.

## Discussion

We have proposed a novel physiologically-plausible mechanism, based on cholinergic modulation of neural membrane excitability, that can account for synaptic renormalization during NREM sleep. We have shown that the dramatic changes in membrane excitability induced by cholinergic modulation, and the resulting changes in network firing patterns, lead to upscaling and downscaling of mean synaptic efficacy. Thus, our results propose a dynamical mechanism for synaptic renormalization that provides a bottom-up framework linking changes in the neuromodulator environment during waking and NREM sleep to changes in neuronal excitability, network activity patterns, and overall renormalization of network connectivity. Simulations of networks with heterogeneous synaptic connection distributions also provided evidence for selective rescaling of particular network connections.

Our simulations showed that high levels of acetylcholine in cortical networks led to asynchronous dynamics, which in turn led to relatively high network potentiation. On the other hand, low levels of acetylcholine resulted in more synchronous network activity and relatively lower overall potentiation. These results are consistent with the prediction of the synaptic renormalization hypothesis that wakefulness (during which ACh is present at high levels in cortex) is associated with global synaptic upscaling, while NREM sleep (during which ACh is present at much lower levels in cortex) is associated with global synaptic downscaling. These results were also robust to noise, changes in network frequency, different network topologies, and various STDP parameters, and they were strengthened by network heterogeneities. Additionally, Fig. 5 shows that extreme concentrations of ACh (either high or low) do not appear necessary to induce the transition from low to high network potentiation–large intervals of 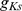 accommodated both states.

The desynchronization of neuronal activity that resulted from high concentration of ACh in our model is expected from PRC theory, since higher ACh induces more Type I-like PRC [25]. Some studies, however, have associated increased ACh with elevated neuronal synchrony. For example, Rodriguez et. al. showed that ACh promoted gamma synchronization in response to light stimuli in cat visual cortex [44]. There have been other studies, however, which have shown the opposite effect. Kalmbach et. al. showed that optogenetically-induced release of ACh by nucleus basalis axons led to an immediate desynchronization of afferent cortical neurons [45], and Metherate et. al. demonstrated that electrical stimulation of the nucleus basalis desynchronized cortical EEG [46]. Thus it seems unclear from the literature exactly how ACh affects neuronal synchronization. One possibility is that ACh enhances synchrony in response to attended stimuli, but has a desynchronizing effect in regions of cortex which are not actively processing attended stimuli. In that case, our model would emphasize endogenous network dynamics over stimulus-evoked activity.

On the other hand, ACh is known to be down-regulated during NREM sleep, when slow wave activity dominates EEG recordings. Such activity is associated with the slow oscillation of thalamocortical neuron membrane potential that results from thalamocortical bistability [47]–[49]. In addition, multiple lines of evidence suggest that slow waves involve the persistent synchronous bursting of cortical neuron populations [5], [50]–[52]. Similar activity patterns were produced in our simulations of low-ACh networks (see Fig. 1d), suggesting that low cholinergic concentration may work in tandem with underlying slow oscillations to facilitate bursting activity. As shown in Fig. 10, this highly synchronous activity resulted in synaptic downscaling relative to the asynchronous activity induced in high-ACh networks.

Fig. 10f also shows how a subset of connections that were highly potentiated following waking (high ACh) remained strong–and were actually even further strengthened–during simulated NREM sleep (low ACh). This effect was obtained through the introduction of a small subset of connections which had larger maximum synaptic strength values than in the rest of the network, providing a possible mechanism for sleep-dependent memory consolidation within the framework of spike-timing dependent plasticity.

While our theory focuses on possible dynamical underpinnings of the renormalization hypothesis, there are many other factors which may contribute to synaptic renormalization. Incoming sensory signals may promote upscaling during wakefulness [4], while downscaling during sleep might be facilitated by the endogenous low-frequency rhythms of slow-wave sleep, which share similar frequency content with the low-frequency stimulation known to induce long-term depression [17], [18]. One recent study suggested that elevated levels of neuromodulators such as noradrenaline and acetylcholine during waking may promote overall synaptic potentiation, while the absence of these same neuromodulators during sleep may modify spike-timing dependent plasticity to favor synaptic depression [21], [22]. Our simplified model focuses upon spike-timing dependent plasticity because we are interested in how network potentiation is affected by alterations in network synchrony, and STDP is the form of plasticity which is most relevant for changes in synchrony. There are, however, many plasticity mechanisms in the brain other than STDP which may also contribute to synaptic renormalization, including the many varieties of homeostatic plasticity [53], [54]. Investigating the interaction between STDP and these other forms of homeostatic plasticity is beyond the scope of this paper.

Our theory hinges on the result that synchronous network activity leads to synaptic downscaling, while asynchronous network activity generates synaptic upscaling. Our analysis of the structure of spike times in pre- and post-synaptic cell pairs indicates that downscaling was due to timing competition between arriving excitatory post-synaptic potentials (EPSPs) within the brief period of synchronous spiking activity. This competition within such a short time window resulted in about half the pre-post pairings falling in the negative portion of the STDP curve and therefore leading to lower network potentiation relative to asynchronous network activity. It has previously been shown that asynchronous neuronal activity leads to increased network potentiation while synchronous activity leads to decreased network potentiation in simulated networks incorporating STDP with propagation delays [55]. Our results show that similar effects can be obtained in networks where synaptic delays are negligible. Additionally, these effects are obtained for completely different and counterintuitive reasons, namely through altered statistics of spike arrival times at post-synaptic cells.

In summary, we have shown that cholinergic modulation can lead to changes in overall network potentiation, and that these changes may be understood in terms of the altered cellular and network dynamics induced by ACh. Further experimental investigation into the possible role of cholinergic modulation in the dynamical underpinnings of synaptic renormalization is clearly required.

## Materials and Methods

### Cortical neuron model

The cortical pyramidal model neuron we employed was motivated by a recent experimental study which showed that in slices of mouse visual cortex, the presence of acetylcholine (ACh) modulated the response properties of cortical neurons as measured by the phase response curve (PRC) [25]. The neuronal PRC tracks the changes in spike timing in response to perturbations of the membrane potential as a function of the phase of the spike cycle at which the perturbation occurs. The presence of ACh and its effects upon neuronal PRCs were shown to be well modeled by varying the maximum conductance 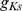 of a slow, low-threshold 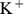-mediated adaptation current from 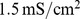 to 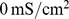 in a Hodgkin-Huxley based neuronal model [26], [56]. We used this model in the current study, and modulated only 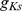 to model the presence or absence of ACh. The model also featured a fast, inward 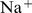 current. The model also includes an inward 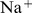 current, a delayed rectifier 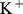 current, and a leakage current. The current balance equation for the 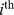 cell was
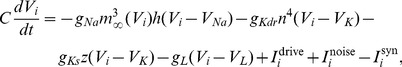
(1)with 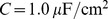, 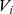 in millivolts, and 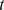 in milliseconds. 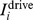 was an externally applied current that was constant for each neuron but Gaussian-distributed across neurons in the network, with a variance set to induce a spread of 1 Hz in the instrinsic neuronal frequencies in the neurons for both high and low levels of cholinergic modulation. The mean of the distribution of 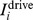 values was 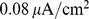 for high-ACh networks and 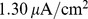 for low-ACh networks (different values were necessary to account for different firing thresholds and frequency-current curves). 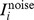 was a Gaussian noise term supplied to each neuron in our study of noise robustness (Fig. 4). This noise was independent from neuron to neuron, but for each individual neuron the noise was correlated over a time scale of 100 ms (the typical inter-spike interval of the slowest-firing neurons). 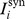 was the synaptic current received by neuron 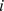.

Activation of the 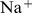 current was instantaneous and governed by the steady-state activation function 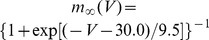. Dynamics of the 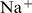 current inactivation gating variable 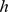 were given by
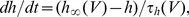
(2)with 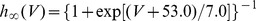 and 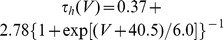. The delayed rectifier 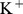 current was gated by 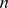, whose dynamics were governed by
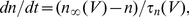
(3)with 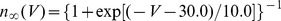 and 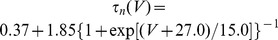. The slow, low-threshold 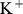 current targeted by cholinergic modulation was gated by 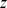, which varied in time according to
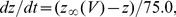
(4)where 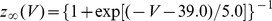.

The slow, low-threshold 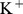 current loosely modeled the muscarine-sensitive M-current observed in cortical neurons. Setting 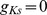 modeled high levels of ACh in cortical networks, and setting 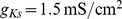 modeled low ACh levels. All other parameter values were the same for both high-ACh and low-ACh networks: 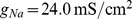, 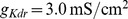, 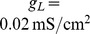, 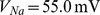, 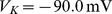, and 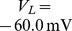.

### PRC calculation

To obtain the phase response curves displayed in Fig. 1, 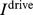 was set to a fixed value to elicit repetitive firing in a single, synaptically isolated neuron, and the model equations were time evolved using a fourth-order Runge-Kutta numerical scheme until the oscillatory period stabilized. Then, using initial conditions associated with the spike peak, brief current pulses were administered at different phases of the oscillation, and the perturbed periods were used to calculate the corresponding phase shifts. The current pulses were administered at 100 equally-spaced time points throughout the period of the neuronal oscillation. The current pulses had a duration of 0.06 ms and an amplitude of 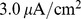 for the high-ACh cortical pyramidal neuron, and a duration of 0.06 ms and an amplitude of 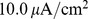 for the low-ACh cortical pyramidal neuron.

### Network simulations

We simulated networks with 800 excitatory neurons and 200 inhibitory neurons. The network connectivity pattern was constructed using the Watts-Strogatz architecture for “small world networks” [35]. Starting with a 1-D ring network with periodic boundary conditions, each neuron was at first directionally coupled to its 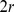 nearest neighbors, and then every connection in the network was rewired with probability 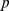 to another neuron selected at random. In this way, 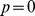 resulted in a locally-connected network and 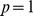 in a randomly connected network. The radius of connectivity 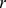 therefore determined the density of connections in the network, while the re-wiring parameter 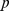 determined the network connectivity structure. Network connectivity 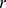 was set to 4 in all simulations except those in Fig. 10 and Fig. 6.

Synaptic current was transmitted from neuron 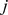 following times 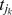 when its membrane voltage breached −20 mV. The synaptic current delivered from neuron 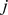 to a synaptically connected neuron 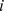 at times 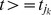 was given by 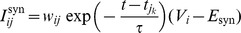, where we used 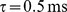 and 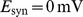 for excitatory synapses and 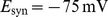 for inhibitory synapses. The total synaptic current to a neuron 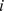 was given by 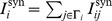, where 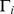 was the set of all neurons presynaptic to neuron 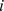. Excitatory synaptic strengths 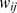 evolved according to an additive STDP rule in which the change in synaptic strength between postsynaptic neuron 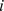 and presynaptic neuron 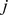 was given by
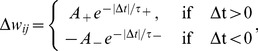
(5)where 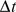 represents the spike time of postsynaptic neuron 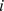 minus the spike time of presynaptic neuron 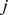. We set 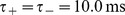 in all our simulations, except in Figs. 7 and 8. We also confined synaptic strength values to the interval 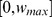, where 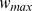 was a parameter that we varied in our simulations. The maximum amount the strength of a synapse could change due to one spike pairing was set by the parameters 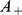 and 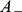, which we set to 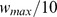 (except for the simulations in Fig. 8). We intentionally chose this value to be rather large so that synaptic strength distributions would equilibrate in a reasonable amount of time.

Simulations were initialized with all synaptic strengths set to 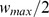, after which the strengths of excitatory synapses evolved freely according to the dynamics of the network (strengths of inhibitory synapses were fixed). After the distribution of synaptic weights had equilibrated (which required longer for low-ACh networks because they fired at lower rates than high-ACh networks; high-ACh network simulations were run for 5,000 ms and low-ACh network simulations were run for 20,000 ms), the overall network potentiation was quantified using the measure
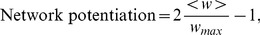
(6)where 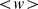 designates the mean of all equilibrium excitatory synaptic strengths. This measure, which is just a scaling of mean synaptic strength, attributed a network potentiation value of +1 to maximally potentiated final synaptic distributions, and a network potentiation value of −1 to maximally *de*potentiated final synaptic distributions. All simulations were numerically integrated in Matlab using a fourth-order Runge-Kutta method with a time step of 0.05 ms.

We quantified phase-synchronization of neuronal firing in our simulations using the mean phase coherence (MPC) measure [57]. This measure quantified the degree of phase locking between neurons, assuming a value of 0 for completely asynchronous spiking and 1 for complete phase locking. Note that high MPC could be attained for locking of phases at *any value*, not just zero. The MPC between a pair of neurons, 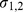, was defined by:
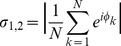
(7)

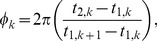
(8)where 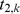 was the time of the 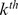 spike of neuron 2, 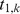 was the time of the spike of neuron 1 that was largest while being less than 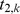, 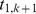 was the time of the spike of neuron 1 that was smallest while being greater than or equal to 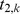, and 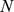 was the number of spikes of neuron 2. The MPC of the entire network was calculated by averaging the mean phase coherence of all neuron pairs, discounting the first half of network activity, in order to capture steady-state network synchronization.

In our simulations exploring network heterogeneity, the network was composed of 1000 neurons (800 excitatory, 200 inhibitory), of which 50 comprised a cluster in which 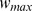 was two times greater than in the rest of the network (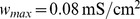 for connections originating from neurons within the cluster, and 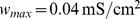 for connections originating from neurons outside the cluster). Connectivity was constructed by initially segregating the cluster from the rest of the network, so that the cluster and the rest of the network formed two disjoint Watts-Strogatz networks, each with a radius of connectivity of 4 and a re-wiring probability of 0.60. The two networks were then coupled by sending three outgoing connections from each cluster neuron to randomly-selected neurons in the rest of the network. Similarly, three outgoing connections were also sent from each neuron in the rest of the network to randomly-selected neurons within the cluster. Simulations were then run in which the network was repeatedly switched between high-ACh and low-ACh states, and the effects on network potentiation were explored. We quantified the network potentiation for all excitatory connections, as before, but also for just the connections which linked the cluster and the rest of the network.
